# Structure-Based Profiling of Potential Phytomolecules with AKT1 a Key Cancer Drug Target

**DOI:** 10.3390/molecules28062597

**Published:** 2023-03-13

**Authors:** Zeenat Mirza, Sajjad Karim

**Affiliations:** 1 King Fahd Medical Research Center, King Abdulaziz University, Jeddah 21589, Saudi Arabia; 2 Department of Medical Laboratory Sciences, Faculty of Applied Medical Sciences, King Abdulaziz University, Jeddah 21589, Saudi Arabia; 3 Center of Excellence in Genomic Medicine Research, King Abdulaziz University, Jeddah 21589, Saudi Arabia

**Keywords:** cancer, biomarkers, AKT1, molecular docking, PI3K/AKT pathway, isoliquiritigenin, shogaol, tehranolide, theophylline

## Abstract

Identifying cancer biomarkers is imperative, as upregulated genes offer a better microenvironment for the tumor; hence, targeted inhibition is preferred. The theme of our study is to predict molecular interactions between cancer biomarker proteins and selected natural compounds. We identified an overexpressed potential molecular target (AKT1) and computationally evaluated its inhibition by four dietary ligands (isoliquiritigenin, shogaol, tehranolide, and theophylline). The three-dimensional structures of protein and phytochemicals were retrieved from the RCSB PDB database (4EKL) and NCBI’s PubChem, respectively. Rational structure-based docking studies were performed using AutoDock. Results were analyzed based primarily on the estimated free binding energy (kcal/mol), hydrogen bonds, and inhibition constant, Ki, to identify the most effective anti-cancer phytomolecule. Toxicity and drug-likeliness prediction were performed using OSIRIS and SwissADME. Amongst the four phytocompounds, tehranolide has better potential to suppress the expression of AKT1 and could be used for anti-cancer drug development, as inhibition of AKT1 is directly associated with the inhibition of growth, progression, and metastasis of the tumor. Docking analyses reveal that tehranolide has the most efficiency in inhibiting AKT1 and has the potential to be used for the therapeutic management of cancer. Natural compounds targeting cancer biomarkers offer less rejection, minimal toxicity, and fewer side effects.

## 1. Introduction

Cancer is one of the major causes of morbidity and mortality worldwide, with approximately 18 million new cancer incidences and 9.6 million fatalities globally based on GLOBOCAN (https://gco.iarc.fr, accessed on 11 October 2022) [1,2]. Unfortunately, it is predicted to increase globally due to unhealthy lifestyles, including smoking, being overweight, and less physical activity. The most common strategies to combat cancer; are (i) for early detection/diagnosis surgery or radiotherapy and (ii) for late/advanced stage detection, which is usually when chemotherapy is applied [3]. When cancer is resistant to radiotherapy and chemotherapy, the prognosis is poor, and side effects severely constrain these standard therapies. Hence, alternative therapeutic cancer targets and natural compounds with higher efficacy and fewer adverse effects need to be explored.

Identifying the overexpressed cancer biomarkers has great potential as it could be suppressed by targeted inhibition. Transcriptomic studies detected multiple differentially expressed genes in different cancer types [4,5,6,7]. A group of genes work together as networks and pathways to regulate cellular activities. However, selecting a key target therapeutic gene is always a challenge. Molecular-targeted therapy for various cancers have been widely applied against identified targets such as cyclin-dependent kinases (CDKs), estrogen receptor (ER), BCR activator of RhoGEF and GTPase (BCR)– ABL protooncogene 1 nonreceptor tyrosine kinase (ABL), epidermal growth factor receptor (EGFR), human epidermal growth factor receptor-2 (HER2), and vascular endothelium growth factor receptor (VEGFR), using selective inhibitors such as imatinib, erlotinib, herceptin, gefitinib, erlotinib, afatinib, and dacomitinib [8,9,10,11]. One of the upregulated genes in several cancer types is AKT serine/threonine kinase 1 (AKT1, also referred to as protein kinase B, PKB). It is catalytically inactive in serum-starved fibroblasts and activated by platelet-derived growth factor via phosphoinositide 3-kinase (PI3K) signaling. It is frequently associated with cell proliferation, growth, survival, angiogenesis, and tumor invasion/metastasis [12,13]. Hyperactivation of AKT protein and alteration in the PI3K/AKT signaling pathway aids tumor progression and promotes chemotherapeutic drug resistance in different cancers [12,13]. Downstream signals of BCR–ABL, EGFR, and HER2 also affect the PI3K/AKT pathway during carcinogenesis [8]. Hence, AKT is rationally considered a therapeutic target for cancer. 

An activating mutation in AKT1 (SNP c.49G > A, p.Glu17Lys) is also strongly associated with Proteus syndrome, an overgrowth progressive disorder caused by a rare genetic mosaicism. The mutation arises during embryonic development and gives rise to overgrowth in a subset of the individual’s cells leading to disproportionate overgrowth, bone abnormalities, intellectual disability, seizures, brain malformations, and deep vein thrombosis, resulting in premature death [14].

Natural phytocompounds, due to their diverse pharmacological effects and the easy availability of medicinal plants, are frequently employed in drug discovery investigations. Previous studies reported in vitro apoptotic activity of curcumin, gallic acid, caffeic acid, and other natural compounds against tumor cells, but with no harmful effect on normal healthy cells or the immune system [15,16,17,18]. Even some modifications in lifestyle can lower the incidence of certain types of cancers; for example, vegetable and fruit-rich diets could include chemoprevention in colorectal cancer. Most chemotherapeutic drugs induce harmful side effects on cytotoxic immune cells, limiting their widespread use in the past. Alternatively, several synthesized and natural compounds have shown cancer chemoprevention potential against cancer targets, including AKT1. Starting with synthesized ligand ML-9 in the year 2000 and NL-71-101, to phytochemicals such as l-quebrachitol, perifosine (NSC639966), capivasertib (AZD5363), and ipatasertib, many substances have been evaluated as AKT inhibitors [19,20,21,22,23]. However, none of these inhibitors passed all the phases of clinical trials, and there is a need to explore new AKT1 inhibitors.

Licorice (*Glycyrrhizae rhizoma*) is a well-known medicinal plant with multiple pharmacological activities, along with anti-cancer effects [2]. Isoliquiritigenin (4,2′,4′-trihydroxychalcone) extracted from *Glycyrrhiza* root is one of the most pharmacologically bioactive compounds that exhibit significant anti-proliferative activity on different cancer cells [24,25,26].

Shogaols (6-, 8-, and 10-shogaol) are constituents of ginger (*Zingiber officinale Roscoe*) and have anti-cancer, anti-inflammatory, and anti-invasive activities, along with being a cure for nausea, vomiting, dyspepsia, pain, cold, and diarrhea [27,28]. The constituent 6-shogaol exhibited antitumor activity in breast, prostate, and colorectal cancer by inhibiting MMP-9, NF-κB, and JNK activation [29,30]. Matrix metalloproteinases code for the endopeptidases responsible for the degradation of the extracellular matrix that enables tumor invasion and metastasis [31]. This extracellular matrix degradation is one of the major reasons for the high cancer mortality [32].

Tehranolide, a bioactive compound of the sesquiterpene lactone group, extracted from *Artemisia diffusa*, shows a wide range of phytomedicinal activities against malaria, microbes, migraine, inflammation, infection, ulcers, and cancer [33]. Tehranolides attribute antitumor effects through the inhibition of proliferation, cell cycle, apoptosis, and alteration of signal transduction pathways [33,34,35,36,37].

Theophylline, a methylxanthine derivative, exhibits antitumor activities by enhancing apoptosis, promoting autophagy, inducing PTEN activity and mTOR inhibition [38]. The PI3K/AKT pathway regulates cell growth and survival [39], and PTEN negatively regulates the PI3K/Akt pathway [40]. PTEN suppression and PTEN-mediated pathways have been reported in tumor development [41,42]. mTOR, a member of the PI3K-related kinases family, forms the mTOR signaling complex that inhibits autophagy and plays a vital role in cell growth and metabolism [43]. Molecular docking is a structure-based method to identify and screen novel compounds of therapeutic interest [44]. The in silico molecular docking-based experiment sorts large compound libraries faster, rationally, and at a significantly reduced cost compared to the traditional drug screening methods [45]. Structure-based methods depend on the information derived from the three-dimensional (3D) structure of a target of interest. They rank ligands based on their structural and electronic complementarity to a given target based on the estimated free energy of binding, inhibition constant (Ki), and observed bonds. Moreover, with the advancements in computer science, structural modeling, simulation graphics, and artificial intelligence (AI), an ever-growing increase has been witnessed with respect to structural, chemical, and biological data available on therapeutic biomarkers [46,47]. Herein, potential effective herbal anti-cancer compounds were evaluated using structure-based molecular docking to predict interactions and suggest a suitable treatment approach as an alternative supplement, or to complement existing therapies. Therefore, we evaluated the inhibitory properties of four phytochemicals, isoliquiritigenin, shogaol, tehranolide, and theophylline, against AKT1, a cancer therapeutic molecular target, using molecular docking.

## 2. Results

### 2.1. Identification of AKT1 as a Potential Biomarker

**Figure 1:**
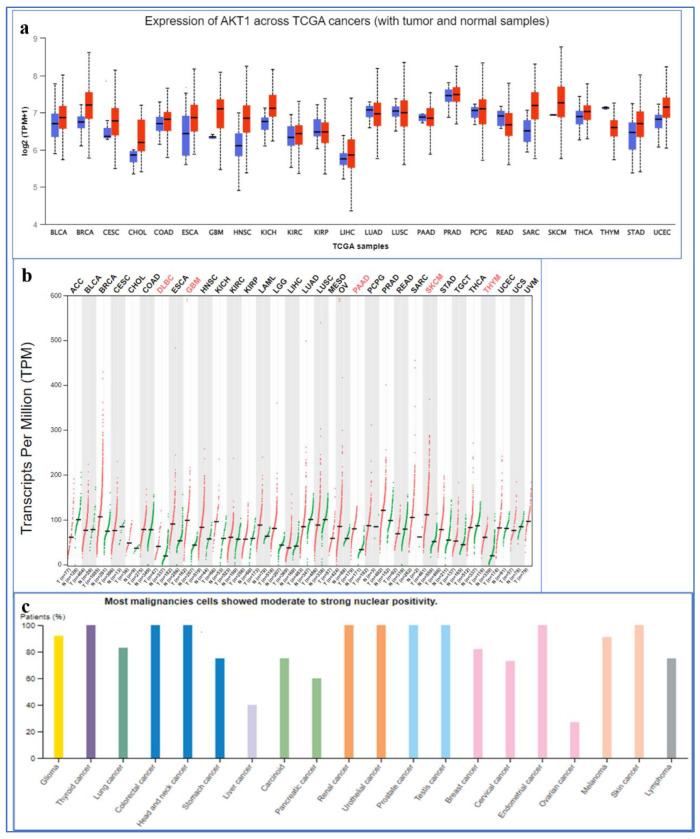
(**a**) AKT1 expression across different cancers according to UALCAN database (blue is for normal and red for tumors), (breast invasive carcinoma (BRCA), cholangiocarcinoma (CHOL), lymphoid neoplasm diffuse large B-cell lymphoma (DLBC), esophageal carcinoma (ESCA), glioblastoma multiforme (GBM), head and neck squamous cell carcinoma (HNSC), kidney chromophobe (KICH), acute myeloid leukaemia (LAML), brain lower-grade glioma (LGG), ovarian serous cystadenocarcinoma (OV), sarcoma (SARC), skin cutaneous melanoma (SKCM) and thymoma (THYM)); (**b**) the gene expression profile (dot plot) across all tumor samples and paired normal tissues according to GEPIA; (**c**) expression of Akt1 in cancer using polyclonal anti-AKT1 antibody HPA002891 (source—Protein Atlas).

**Figure 2:**
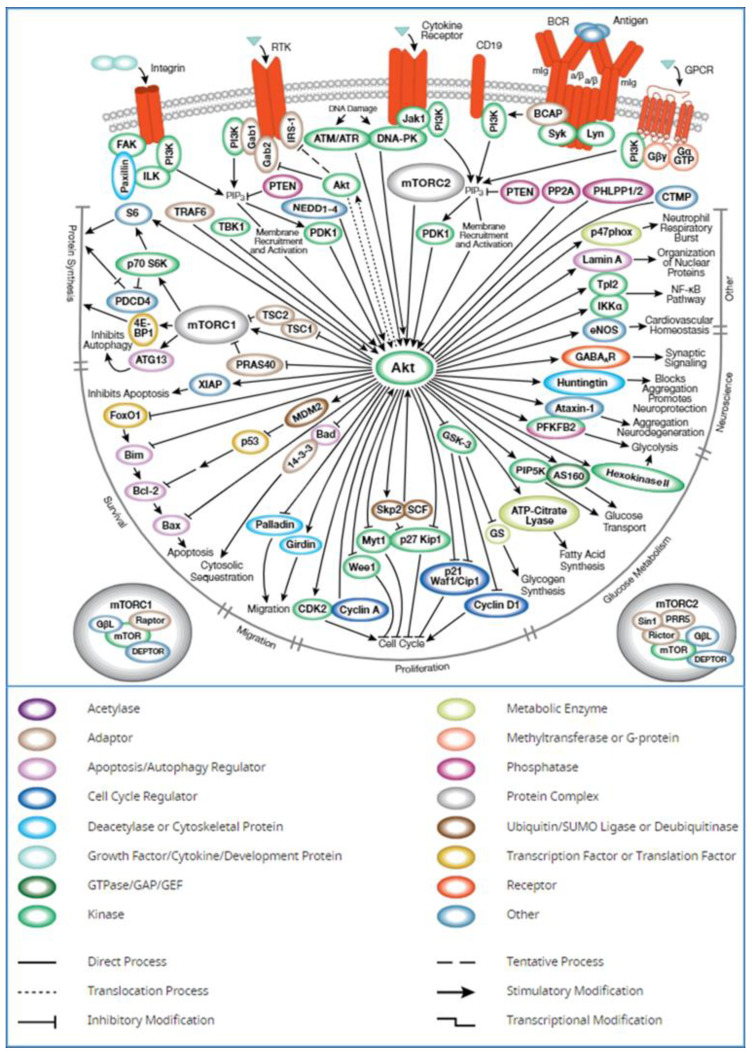
PI3k/AKT signaling pathway. Illustration reproduced courtesy of Cell Signaling Technology Inc. (www.cellsignal.com, accessed on 30 October 2022).

It is evident from the UALCAN database (TCGA) that expression of AKT1 is higher in cancer as compared to normal samples (Figure 1a). According to GEPIA, significant differential overexpression of AKT1 was seen in invasive breast carcinoma, cholangiocarcinoma, lymphoid neoplasm diffuse large B-cell lymphoma, esophageal carcinoma, glioblastoma multiforme, head and neck squamous cell carcinoma, kidney chromophobe, acute myeloid leukemia, brain lower-grade glioma, ovarian serous cystadenocarcinoma, sarcoma, skin cutaneous melanoma, and thymoma (Figure 1b). The human protein atlas reports high consistency amongst antibody staining and AKT1 RNA expression data. Also, moderate to high cytoplasmic and nuclear staining was observed in malignant melanomas, gliomas, and breast and prostate cancers, albeit with low tissue and cancer specificity (Figure 1c). One of the most notably over-activated intracellular pathways in several cancers is the PI3K/AKT signaling pathway, which acts on multiple downstream target proteins, leading to carcinogenesis, proliferation, invasion, and metastasis (Figure 2).

### 2.2. Protein Structure Analysis

The PDB 4EKL was selected as the receptor and active site, and other important residues were enlisted for AKT1. Schrodinger’s protein preparation tool and SiteMap module were used to identify the potential ligand binding sites. The binding site was deep, consisting primarily of hydrophobic amino acid residues. The potential binding site properties and druggability were also predicted using DoGSiteScorer based on the 3D structure of the protein and splitting them into sub-pockets. A higher score of 0.83 was predicted, indicating that the pocket is estimated to be druggable. Significant amino acid residues, primarily hydrophobic, are present in the binding site include Leu156, Gly157, Lys158, Gly159, Gly162, Val164, Ala177, Thr211, Met227, Glu228, Tyr229, Ala230, Glu234, Glu278, Met281, Thr291, and Phe438.

### 2.3. Ligand Selection and Preparation

**Figure 3:**
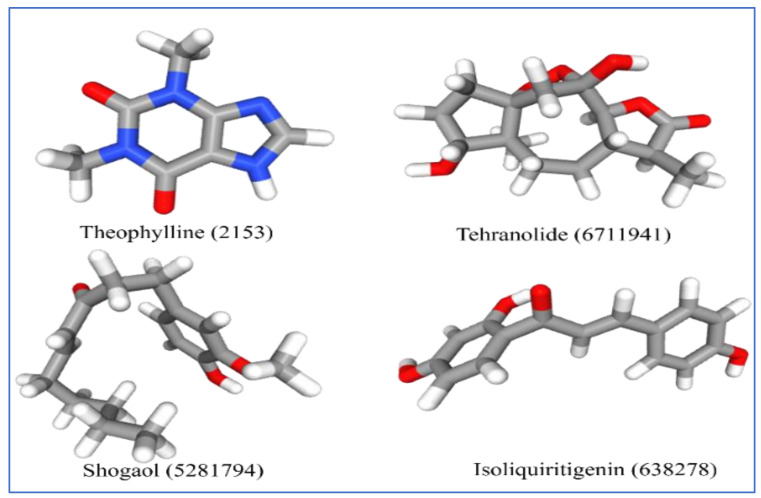
Three-dimensional structure of selected inhibitors with their PubChem CID.

The selected four ligands were prepared for docking. The 3D structure of the ligand compounds is shown in Figure 3.

### 2.4. Molecular Docking and Analysis of the Complex

Binding free energy, a sum of all the intermolecular interactions between the ligand and the target, and inhibition constant (Ki) were calculated by the AutoDock tool. Ki, the inhibitor constant, is an indication of how potent an inhibitor is; it is the concentration required to produce half maximum inhibition. The lower the Ki for a particular drug at a specific receptor, the stronger its binding affinity. This is because the lower Ki means that the drug can occupy 50% of those receptors even when the drug is present in a lower concentration. Hence, the smaller the Ki, the greater the binding affinity and the smaller amount of medication needed in order to inhibit the activity of that enzyme.

**Figure 4:**
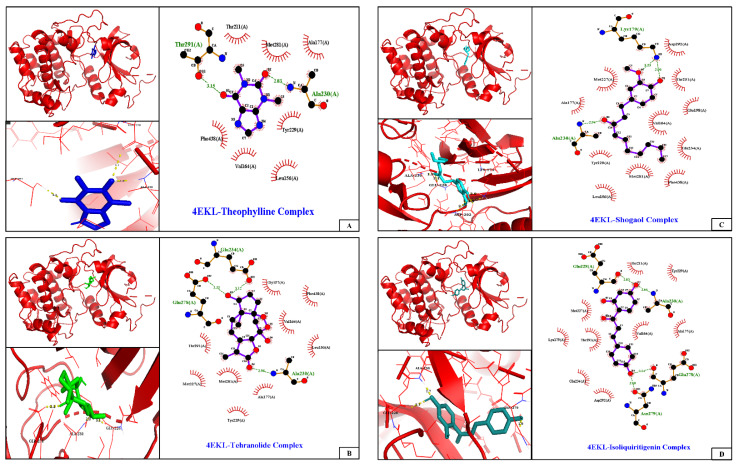
Three-dimensional structure of AKT1 protein (4EKL) with its active site drawn by PyMOL v2.5.0) and the ligand–protein interaction 2D plot for theophylline (**A**), tehranolide (**B**), shogaol (**C**) and isoliquiritigenin (**D**) drawn using Ligplot+ v2.2. H-bonds are designated by dashed lines between the atoms involved, while hydrophobic interactions are depicted by radiating arc towards the ligand atoms they contact.

In the present case, when we consider all the compounds Ki in same unit nM, then we observe that the compounds arranged in order of inhibition: tehranolide > isoliquiritigenin > shogaol > theophylline. AutoDock predicted tehranolide (binding energy −9.22 kcal/mol, Ki 173.21 nM) forming three hydrogen bonds with Ala230, Glu234, and Glu278 as the best docking interacting ligand against AKT1, followed by isoliquiritigenin, shogaol, and theophylline (Table 1, Figure 4). No unfavorable interactions were observed. Other interactions primarily mean hydrophobic interactions, Van der Waals interactions, and sometimes pi–pi ring stacking and halogen bonding. The drug-binding domain of AKT1 is the hydrophobic cavity situated at the lower interface between the N- and C-lobes of the kinase domain.

### 2.5. Analysis of ADMET Properties

OSIRIS, a drug discovery informatics platform, was used for toxicity risk assessment through cLogP prediction, solubility prediction, fragment-based drug-likeness prediction, irritant, and overall drug score (Table 2). SwissADME, a tool to evaluate pharmaceutical properties, was used to compute the ligands’ physical and medicinal chemical properties (Table 3). The drug score combines drug-likeness, cLogP, logS, molecular weight, and toxicity risks into one value ranging from 0 to 1, which may help to find the compound’s overall potential to qualify for a drug.

## 3. Discussion

Cancer is a devastating disease globally, and causes millions of deaths across the world despite existing treatments, with frequent resistance to chemotherapy and other treatment methods. Therefore, researchers are exploring natural phytochemicals with potential anti-cancer activity or providing novel lead pharmacological compounds for better drug synthesis, as natural resources have provided medical solutions to different diseases in the past [48]. Cancer is a proliferation disorder and could be regulated by a network of genes, transcription factors, and signaling pathways. Detecting such molecular targets and applying anti-cancer compounds/agents could inhibit uncontrolled cell division and potentially be used for rational tumor therapy [35,48]. The altered expression of kinases results in many cellular abnormalities that can lead to cancer and are, therefore, thought to be a suitable therapeutic target. Overexpression of the AKT-activated PI3K/AKT signaling pathway is a common molecular attribute of several cancers [49]. Hence, targeting AKT seems a potential therapeutic option, and direct inhibition of AKT kinase activity can attenuate cancer growth. A myriad of phytochemicals offers anti-cancer properties by regulating cell cycle, cell death, angiogenesis, and metastasis. To date, both synthetic and natural compounds have been explored, and few are presently under preclinical investigations and clinical development. Different phytochemicals utilize different mechanisms to regulate the cell cycle, antioxidant stress, apoptosis, and immune system.

We computationally investigated a few phytochemical flavonoids to establish their mechanism of action and potential to inhibit AKT1, a therapeutic cancer biomarker. All the four selected compounds were previously known to be anti-carcinogenic and we chose them to explore their probable efficacy in inhibiting AKT1 specifically. Molecular docking, a computational method for mimicking and simulating biological interactions, helped us to determine the best-interacting ligand molecule with the maximum affinity to the specified AKT1 target. Phytocompounds (isoliquiritigenin, shogaol, tehranolide, theophylline) that have an antitumor effect with lower toxicity and side effects were selected and docked with the indicated therapeutic target, AKT1. Hydrophobic residues line the binding cavity. The displacement of protein-bound water molecules into the bulk solvent via interactions, particularly H-bonds, increases ligand-binding affinity. The overexpression of phosphorylated AKT (p-AKT) is a significant flaw in several tumor types. p-AKT protein can impede apoptosis by inhibiting the function of Bax protein. Theophylline was initially used as a bronchodilator and has been shown lately to directly inhibit phosphoinositide-3-kinases, with high potency [50]. Theophylline derivatives can trigger apoptosis by suppressing AKT phosphorylation [51]. Alternatively, theophylline can also suppress serine/arginine-rich splicing factors (SRSF3)-p53 pathway and synergistically act with caffeine to downregulate the amount of wild-type SRSF3. It has the ability to switch p53 from an α-isoform into a β-isoform mediated through the SRSF3-dependent splicing pathway [52]. Theophylline has been reported to enhance the toxicity of doxorubicin to tumor cells and when it is used in combination with gemcitabine or cisplatin, it can induce apoptosis in a variety of tumor cells [51].

Studies have shown strong antitumor activity of flavonoid (6-shogaol, a component derived from dry ginger roots) against the skin, ovary, liver, prostate, and breast cancer without harming healthy cells [49,53,54,55]. Shogaol suppresses proliferation by inhibiting the PI3K/AKT/mTOR network by directly inhibiting AKT1 and AKT2. It binds AKT at an allosteric location at a lower interface amid the N- and C-lobes of the kinase domain [49]. Shogaol shows hydrophobic interactions with several residues more prominently with Ala177 at 3.9 Å, as identified by PLIP.

Isoliquiritigenin, a flavonoid and phenolic secondary metabolite, is found in several foods such as licorice (*Hibiscus sabbariffa*), jicamas (*Pachyrhizus erosus*), red peppers (*Capsicum annuum*), Chinese broccoli (*Brassica alboglabra*), and squash berries (*Viburnum edule*) and has shown antitumor activities [56,57,58]. Suppression of cyclin D1 and the PI3K/AKT pathway by isoliquiritigenin exhibits antitumor properties in Hep3B cells, a liver cancer cell line [57]. In vitro study shows isoliquiritigenin interaction with gamma-aminobutyric acid type-B receptor subunit 1 and targeting of miR-301b/LRIG1 signaling pathways causes suppression of melanoma growth [58]. It was also found to inhibit the angiogenic AKT signaling in glioma [56].

Tehranolide, a natural sesquiterpene lactone, has been shown to induce the G0/G1 arrest and apoptosis and inhibits proliferation of MCF-7 breast cancer cells through ROS production, pAKT downregulation, and modulating the PI3K/AKT signaling pathway [35]. Tehranolide was found to inhibit cancer cell growth by PDE1 inhibition of phosphodiesterase 1 and activation of cAMP-dependent protein kinase A [34]. A persistent antitumor immunity against cancer was reported in turmeric mice treated with tehranolide [59]. The best affinity for AKT1 and inhibition is shown by tehranolide.

**Figure 5:**
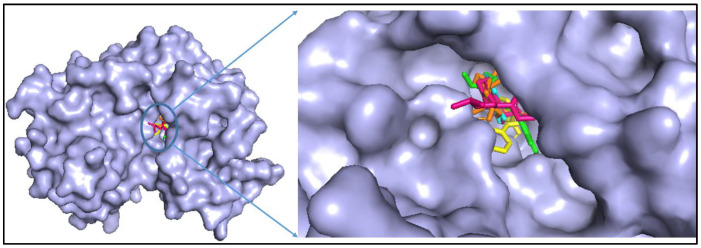
Surface diagram with docked ligands in the active site. The surface representation (PDB: 4EKL) shows the binding mode of the original crystallized ligand GDC0068 (pink) and the docked ligands isoliquiritigenin (yellow), theophylline (cyan), tehranolide (orange), and shogaol (green). Figure made using PyMOL v2.5.0.

cLogP and solubility values are estimated by applying an atom-based increment system. The drug score value combines all other predictions into one grand total. Lipophilicity is depicted as consensus Log Po/w, which is the average of all the five predictions (iLOGP, XLOGP3, WLOGP, MLOGP, SILICOS-IT). Isoliquiritigenin and shogaol are predicted to permeate the blood–brain barrier passively. Similar binding site residues were identified as the co-crystallized ligand in the PDB structure. Investigation of docking results, drug-likeness properties, and toxicity prediction results indicate that all the selected ligands show inhibition and bind at the same hydrophobic pocket (Figure 5), all have an optimum bioavailability score and drug-likeness, and, hence, could be a supplementation option for cancer therapy. Future perspectives of the therapeutic use of isoliquiritigenin, shogaol, tehranolide, and theophylline need to be explored further.

Natural compounds targeting AKT1 can be employed to control pathways with anti-cancer effects. The present computational study is a prediction for evaluating the specific interactions between the chosen natural ligands and AKT1, an overexpressed protein in cancer. This study can potentially aid in developing effective and selective AKT1 inhibitors to control cancer. In vitro enzyme inhibition assays and in vivo cell-based kinase assays can be employed for validation of results, while RT-PCR and microarray-based expression studies can be performed to assess the effect of inhibitors.

## 4. Materials and Methods

### 4.1. Identification of Biomarker

We explored ‘The Cancer Genome Atlas’ (TCGA) gene analysis tool in the UALCAN database (http://ualcan.path.uab.edu/analysis.html, accessed on 4 November 2022), which facilitates tumor subgroup gene expression and shows the percentage of the gene expression’s rate in cancer versus normal [60]. Gene expression profiling interactive analysis (GEPIA) (http://gepia.cancer-pku.cn/index.html, accessed on 4 November 2022) was also used to explore the TCGA dataset and cross-check our hypothesis regarding AKT1 overexpression in cancer. Furthermore, to estimate the antibody-based expression of AKT1 in cancer, we used Protein Atlas (https://www.proteinatlas.org/, accessed on 8 November 2022), which maps all the human proteins by integrating technologies such as antibody-based imaging, proteomics, transcriptomics, and systems biology [61].

### 4.2. Selection and Preparation of Protein Target

The 3D molecular structure of AKT1 determined through X-ray crystallography at 2.0 Å was already available in the RCSB’s protein data bank. The AKT1 structure (source: human) was retrieved in .pdb format (PDB ID: 4EKL), visualized, and further prepared for the docking process. Receptor grid generation and protein preparation was performed. The missing side chain residues and missing loops were filled, bond orders were automatically assigned based on distances, and grid generation was performed based on the reported binding site residues. Furthermore, the potential binding pocket of the protein was also identified using a grid-based method, DoGSiteScorer at Proteins*Plus*, a structure-based modeling support server (https://proteins.plus/, accessed on 11 November 2022) [62].

### 4.3. Ligand Preparation

Potential ligands were selected from the virtual screening method of GLIDE and confirmed by a search of the literature. Their structures were retrieved from the NCBI’s PubChem database [Isoliquiritigenin (PubChem CID: 638278), Shogaol (PubChem CID: 5281794), Tehranolide (PubChem CID: 6711941), Theophylline (PubChem CID: 2153)] in .sdf/.mol2 file format and ligand library preparation were completed using LigPrep. Ligands were converted into 3D and various tautomers/ionization states/stereoisomers were generated.

### 4.4. Molecular Docking and Protein–Ligand Interaction

The molecular docking of ligands into the binding sites of AKT1, a cancer drug target, was performed using AutoDock. The molecular docking results were analyzed quantitatively, and the poses of various ligands with low binding scores were filtered out. The resulting molecular docking scores representing the binding energies in kcal/mol were enlisted. Docked poses were analyzed, and compounds were ranked as per the estimated binding free energy. The docked protein–ligand complexes were analyzed via illustrations of protein–ligand complex prepared using PyMOL Molecular Graphics System, Version 2.5.0 Schrödinger, LLC. For further analysis of the docked protein–ligand interaction using LigPlot+ v2.2 was used to examine the polar and hydrophobic interactions between the protein and ligand. Protein–ligand interaction profiler (PLIP) was further used to identify non-covalent interactions between protein macromolecules and their docked ligands to obtain detailed information on binding characteristics [63].

### 4.5. Prediction of ADME and Toxicity

The absorption, distribution, metabolism, and excretion (ADME) parameters, physicochemical, and pharmacokinetic properties, and drug-like nature of the particular ligands were predicted using SwissADME (http://www.swissadme.ch/, accessed on 14 November 2022) [64]. It also indicates compliance and drug propensity based on Lipinski, Ghose, Veber, Egan, and Muegge filters. Toxicity and other drug-relevant properties were predicted using OSIRIS Property Explorer (https://www.organic-chemistry.org/prog/peo/, accessed on 14 November 2022).

## 5. Conclusions

The primary causes of different malignancies are the interactions among several intrinsic and extrinsic variables. Targeting these anomalies could be significant for the diagnosis and treatment of cancer, as dysregulation of multiple genes and signaling pathways is a well-established phenomenon during various malignancies. The AKT1 enzyme has been found to be overexpressed in various malignancies and, hence, is considered a prospective target for the development of anti-cancer therapeutics. Long-term phytochemicals-based chemoprevention can potentially avert, inhibit, or even reverse carcinogenesis, and has gained prominence, especially in the present times when the use of natural health products and complementary and alternative medicine are increasing. This study reports four natural phytocompounds with strong binding affinities and inhibitory effects against AKT1, indicating their potential as anti-cancer therapeutics. However, further in vitro and in vivo studies followed by a toxicological study of these molecules in a dose-dependent manner prior to clinical trials would be required to substantiate their therapeutic and pharmacological importance. This study can pave the way for kinase-targeted cancer therapies in the future.

## Figures and Tables

**Table 1:** Docking results of AutoDock showing binding energy, inhibition constant, and interacting residues for ligands (tehranolide, shogaol, isoliquiritigenin, theophylline).

Ligands	Binding Energy (kcal/mol)	Inhibition Constant (Ki)	Interacting Residues	
			H-Bonds (Distances)	Others
Tehranolide (6711941)	−9.22	173.21 nM	Ala230 (2.0 Å), Glu234 (2.2 Å), Glu278 (3.33 Å)	Leu156, Gly157, Val164, Ala177, Met227, Tyr229, Met281, Thr291, Phe438
Shogaol (5281794)	−8.19	992.48 nM	Lys179 (2.96), Glu228 (3.4 Å), Ala230 (1.9 Å)	Leu156, Val164, Ala177, Glu198, Met227, Tyr229, Glu234, Met281, Thr291, Asp292, Phe438
Isoliquiritigenin (638278)	−8.29	841.68 nM	Glu228 (2.1 Å), Ala230 (1.7 Å), Glu278 (3.14 Å), Asn279 (1.9 Å), Thr291 (2.1 Å)	Val164, Ala177, Lys179, Thr211, Met227, Tyr229, Glu234, Asp292
Theophylline (2153)	−7.26	4.73 µM	Glu228 (3.1 Å), Ala230 (1.9 Å), Thr291 (2.2 Å)	Leu156, Val164, Ala177, Thr211, Tyr229, Met281, Phe438

**Table 2:** Toxicity risk assessment of ligands (theophylline, tehranolide, shogaol, and isoliquiritigenin) using OSIRIS platform.

Compound Property	Theophylline	Tehranolide	Shogaol	Isoliquiritigenin
cLogP	−0.31	1.18	4.33	2.27
Solubility	−1.48	−2.81	−3.42	−2.95
Molecular weight	180.0	298.33	276.0	256.0
Drug-likeness	2.51	0.71	−14.4	0.76
Mutagenic	High	None	High	High
Tumorigenic	High	High	None	None
Irritant	None	None	None	Medium
Reproductive effects	High	None	None	None
Drug-score	0.2	0.45	0.22	0.359

**Table 3:** Computed physical, chemical, pharmacokinetics, and drug-likeness properties of ligands (theophylline, tehranolide, shogaol, and isoliquiritigenin) using SwissADME.

Compound Property	Isoliquiritigenin 638278	Shogaol 5281794	Tehranolide 6711941	Theophylline 2153
**General Properties**				
**Structure**	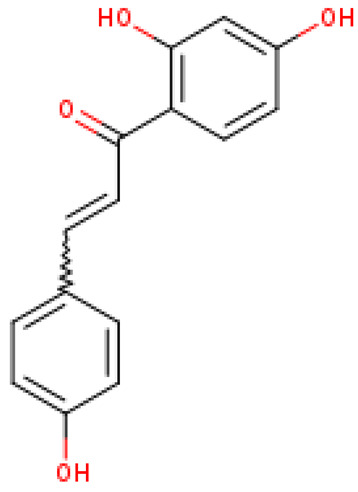	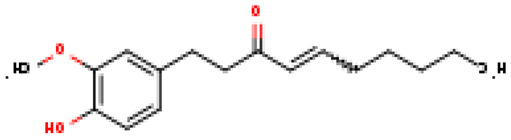	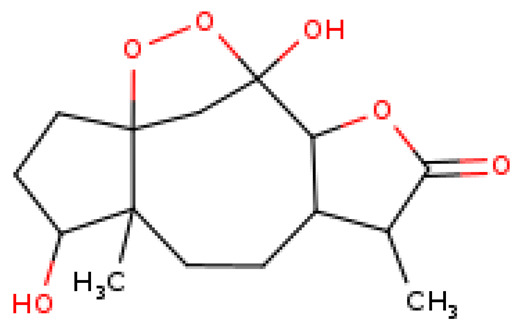	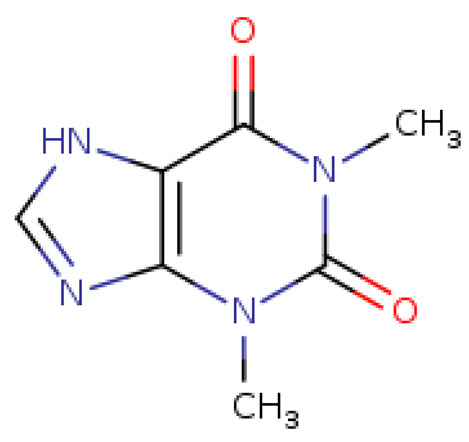
**Bioavailability radar**	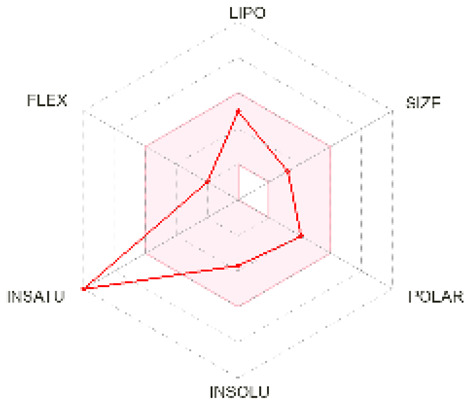	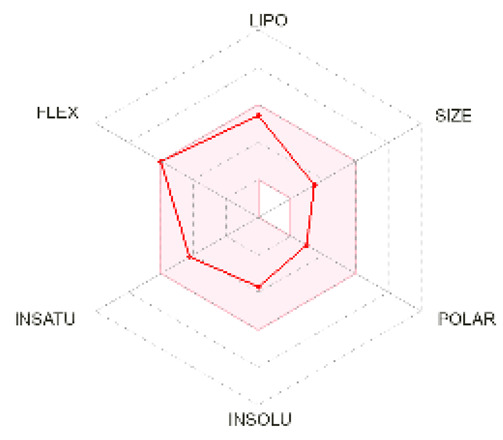	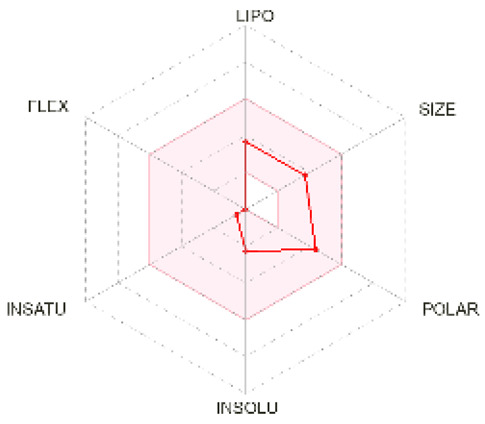	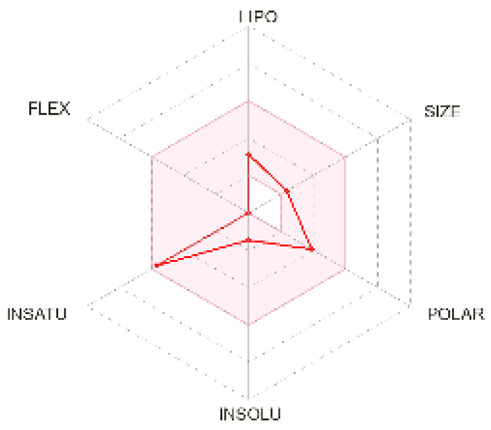
**Smiles**	C1=CC(=CC=C1C=CC(=O)C2=C(C=C(C=C2)O)O)O	CCCCCC=CC(=O)CCC1=CC(=C(C=C1)O)OC	CC1C2CCC3(C(CCC34CC(C2OC1=O)(OO4)O)O)C	CN1C2=C(C(=O)N(C1=O)C)NC=N2
**Physiochemical Properties**				
**Formula**	C_15_H_12_O_4_	C_17_H_24_O_3_	C_15_H_22_O_6_	C_7_H_8_N_4_O_2_
**Molecular weight**	256.25 g/mol	276.37 g/mol	298.33 g/mol	180.16 g/mol
**XLogP3-AA**			0.9	
**Rotatable bonds**	3	9	0	0
**H-bond acceptors**	4	3	6	3
**H-bond donors**	3	1	2	1
**Topological polar surface area**	77.76 Å^2^	46.53 Å^2^	85.22 Å^2^	72.68 Å^2^
**Lipophilicity** **(Consensus Log P_o/w_)**	2.37	3.76	1.20	−0.19
**Water solubility**	Soluble	Moderately soluble	Soluble	Soluble
**Pharmacokinetics**				
**GI absorption**	High	High	High	High
**BBB permeant**	Yes	Yes	No	No
**P-gp substrate**	No	No	Yes	No
**CYP1A2 inhibitor**	Yes	Yes	No	No
**CYP2C19 inhibitor**	No	Yes	No	No
**CYP2C9 inhibitor**	Yes	No	No	No
**CYP2D6 inhibitor**	No	Yes	No	No
**CYP3A4 inhibitor**	Yes	No	No	No
**Log Kp (skin permeation)**	−5.61 cm/s	−5.15 cm/s	−7.45 cm/s	−7.41 cm/s
**Drug-likeness**				
**Lipinski**	Yes; 0 violation	Yes; 0 violation	Yes; 0 violation	Yes; 0 violation
**Ghose**	Yes	Yes	Yes	No; 1 violation: WLOGP < −0.4
**Veber**	Yes	Yes	Yes	Yes
**Egan**	Yes	Yes	Yes	Yes
**Muegge**	Yes	Yes	Yes	No; 1 violation: MW < 200
**Bioavailability score**	0.55	0.55	0.55	0.55
**Synthetic accessibility**	2.52	2.51	5.65	1.87

## Data Availability

All the data have been provided in the manuscript.
